# Targeting the powerhouse: the mitochondrial perspective on gentamicin-induced kidney injury

**DOI:** 10.1007/s00204-026-04310-5

**Published:** 2026-02-07

**Authors:** Busra Korkut Celikates, Sinem Ilgin, Melis Umay Yilmaz, Ozlem Atli Eklioglu

**Affiliations:** Department of Pharmaceutical Toxicology, Faculty of Pharmacy, Anadolu University, 26470 Eskisehir, Turkey

**Keywords:** Gentamicin, Nephrotoxicity, Mitochondrial dysfunction, Reactive oxygen species, Mitochondrial permeability transition pore, Oxidative stress

## Abstract

Gentamicin (GEN), an aminoglycoside antibiotic, induces nephrotoxicity primarily via mitochondrial dysfunction. This review summarizes mechanisms including reactive oxygen species (ROS) overproduction, mitochondrial DNA (mtDNA) damage, impairment of oxidative phosphorylation, and mitochondrial permeability transition pore (mPTP) activation. These mitochondrial alterations lead to adenosine triphosphate (ATP) depletion, apoptosis, and renal injury. In addition to apoptotic pathways, necrotic cell death can also be triggered, further aggravating kidney damage. Furthermore, GEN has been reported to directly interfere with mitochondrial ribosomes and gene expression, highlighting mitochondria as both targets and amplifiers of cellular toxicity. Therapeutic approaches targeting mitochondrial integrity, including antioxidants and mitochondrial transplantation, demonstrate potential nephroprotection. Additional strategies such as mPTP, stimulation of mitochondrial biogenesis, and pharmacological modulators of mitochondrial respiration have also shown promise in experimental studies. Understanding mitochondrial mechanisms underlying gentamicin-induced renal injury is crucial for developing targeted therapeutic strategies. A more comprehensive knowledge of mitochondrial regulation, organelle crosstalk, and early biomarkers of dysfunction will facilitate translation into clinical practice. Overall, preserving mitochondrial function represents a promising avenue for reducing nephrotoxicity while maintaining the antibacterial efficacy of GEN.

## Introduction

Mitochondria, often referred to as the “powerhouse of the cell,” generate adenosine triphosphate (ATP), the principal energy currency required for cellular survival and function in eukaryotic cells. In addition to energy metabolism, mitochondria are pivotal in numerous cellular processes, including signaling, differentiation, cell cycle regulation, and programmed cell death (Anderson et al. 2019; Smith et al. 2012; Wu et al. 2018). Disruption of these processes due to mitochondrial dysfunction is implicated in diverse human pathologies (Smith et al. 2012). Awareness of the contribution of mitochondrial dysfunction to both rare inherited disorders and common conditions—such as cancer, obesity, diabetes, and neurodegenerative diseases—has significantly increased in recent decades (Li et al. 2020; Meyer et al. 2018; Orsucci et al. 2021). Although mitochondrial genome mutations are rare, affecting approximately one in 4,300 individuals (Meyer et al. 2018; Orsucci et al. 2021), many more are impacted by diseases associated with secondary mitochondrial dysfunction.

Drug-induced mitochondrial toxicity has emerged as a major concern in pharmacology and toxicology, as mitochondria are frequent off-targets of xenobiotics (Lin et al. 2021; Vuda and Kamath 2016). This susceptibility arises from their high energy demand, abundance of redox-active enzymes, and unique membrane composition rich in cardiolipin, which facilitates drug accumulation. Many therapeutic agents—including antidiabetic drugs (thiazolidinediones, fibrates, biguanides), statins, certain antidepressants (serotonin antagonist and reuptake inhibitors), non-steroidal anti-inflammatory drugs, antibiotics (fluoroquinolones, macrolides), and anticancer agents (kinase inhibitors, anthracyclines)—can adversely affect mitochondrial function (Will et al. 2019). These toxicities occur through direct inhibition of electron transport chain (ETC) complexes, disruption of mitochondrial DNA (mtDNA) transcription and ATP synthesis, or indirect mechanisms such as induction of oxidative stress and mitochondrial membrane depolarization (Battogtokh et al. 2018; Juan et al. 2021; Vuda and Kamath 2016). Detecting these effects during preclinical studies is challenging, as standard animal models often lack the genetic diversity, environmental stressors, and histopathological sensitivity needed to reveal mitochondrial failure (Will et al. 2019). Drug-induced mitochondrial toxicity remains a leading cause of preclinical attrition, post-market drug withdrawal, and regulatory black box warnings (Wallace and Starkov 2000).

## Targets for drug-induced mitochondrial dysfunction

Mitochondria possess a dual-membrane system: the outer membrane and the highly selective inner membrane, which encloses the mitochondrial matrix. These membranes differ in lipid and protein composition and play distinct roles in mitochondrial dynamics, mitophagy, and apoptosis (Caito and Aschner 2015). Enzymes embedded in the outer membrane integrate mitochondrial metabolism with broader cellular processes (Guo et al. 2013; Wallace and Starkov 2000). Due to its porosity, the outer membrane does not sustain an electrochemical gradient. By contrast, the inner membrane forms a tight diffusion barrier, selectively allowing passage of ions and metabolites via dedicated carrier proteins (Halestrap 2009; Kühlbrandt 2015; Wang and Hekimi 2013). Embedded within this membrane are the protein complexes of the ETC responsible for oxidative phosphorylation. Its unique lipid composition, rich in cardiolipin and nearly devoid of cholesterol, facilitates proton gradients and also predisposes mitochondria to drug accumulation and injury (Szeto 2014; Wallace and Starkov 2000). The inner membrane maintains an electrochemical membrane potential (~ 180 mV) generated by proton pumps (complexes I, III, and IV), which drives ATP synthesis and regulates calcium homeostasis and reactive oxygen species (ROS) generation (Kühlbrandt 2015; Nicholls 2004; Zorova et al. 2018).

A critical structure associated with mitochondrial permeability is the mitochondrial permeability transition pore (mPTP). The inner membrane contains numerous transmembrane proteins, and under pathological conditions—such as calcium overload, oxidative stress, or adenine nucleotide depletion—these proteins can assemble into the mPTP, a multiprotein complex whose opening leads to water influx, mitochondrial swelling, outer membrane rupture, and ultimately apoptosis or necrosis (Halestrap 2009; Marroquin et al. 2014; Pessayre et al. 2010). Drugs may disrupt mitochondrial integrity either by inducing mPTP opening or by inhibiting specific carrier proteins and ETC complexes, thereby compromising ATP production and redox balance (Szeto 2014; Wallace and Starkov 2000).

The mitochondrial matrix contains many circular mtDNA that do not contain introns and histones and all the enzymes and transcription factors necessary for its replication, transcription, translation, and repair (Fromenty 2019). mtDNA constitutes approximately 0.25% of the cell’s genetic structure and encodes only 13 mitochondrial respiratory chain proteins (Fromenty 2019; Dykens et al. 2008). All other mitochondrial proteins, numbering approximately 1700, are encoded by nuclear DNA (Fromenty 2019). It is noteworthy that mitochondria are equipped with DNA repair pathways similar to those elucidated for the nuclear genome, such as the base excision repair system, mismatch repair-like activity, non-homologous end joining, and potentially homologous recombination (Sharma and Sampath 2019). Normal and mutated mtDNA copies can coexist within the same tissue in specific pathological conditions like mitochondrial diseases and the aging process. Importantly, most disorders originating from mtDNA mutations are functionally recessive, requiring a high mutant load for clinical pathology to manifest. Severe mitochondrial dysfunction and adverse events typically correlate with a decline in mtDNA copies to levels below 20–40% of their baseline quantities. Under such circumstances, the residual functional mtDNA copies within mitochondria prove inadequate for encoding an adequate complement of their respective proteins, leading to compromised oxidative phosphorylation (Fromenty 2019). Drugs can induce damage to mtDNA through the inhibition of mtDNA replication or by provoking oxidative damage within mtDNA via the excessive generation of ROS (Fromenty 2019; Roubicek and Souza-Pinto 2017). Additionally, drugs can interfere with mtDNA translation by interacting with mitochondrial ribosomes (Fromenty 2019).

### Cellular energy production

In its high-energy form, ATP is essential for maintaining cellular homeostasis and viability (Kim and Choi 2021). Within the cytoplasm, glycolysis metabolizes glucose to produce two pyruvate molecules, generating ATP and nicotinamide adenine dinucleotide (NADH) (Chaudhry and Varacallo 2023; Yang et al. 2021). Pyruvate is subsequently transported into the mitochondria and oxidized to acetyl-coenzyme A (acetyl-CoA), which enters the tricarboxylic acid (TCA) cycle. The TCA cycle produces additional NADH, flavin adenine dinucleotide (FADH_2_), and CO_2_, driving downstream oxidative phosphorylation (Alabduladhem and Bordoni 2024; Haddad and Mohiuddin 2024; Yang et al. 2021). Acetyl-CoA generated via the β-oxidation pathway of fatty acids also contributes to the TCA cycle (Houten et al. 2016; Ruiz-Sala and Peña-Quintana 2021). Electrons stored in NADH and FADH_2_ are transferred to oxygen via the mitochondrial ETC, producing ATP through a series of redox reactions known as oxidative phosphorylation (Ahmad and Cox 2014; Deshpande and Mohiuddin 2023; Yang et al. 2021).

Drug-induced toxicity can occur when this energy-generating system is disrupted. Inhibition of electron flow through ETC complexes I–IV is a well-established mechanism, either via direct inhibition of protein subunits or by electron diversion from natural carriers such as ubiquinone or cytochrome c (Krähenbühl 2001; Scatena 2012). Inhibition of enzymes involved in β-oxidation halts fatty acid breakdown to acetyl-CoA, either directly or indirectly through ETC inhibition, leading to depletion of required cofactors and impaired mitochondrial energy metabolism (Betiu et al. 2022; Fabbrini and Magkos 2015; Houten et al. 2016; Ruiz-Sala and Peña-Quintana 2021).

### Mitochondrial dysfunction in gentamicin-induced nephrotoxicity

Mitochondrial dysfunction is implicated in both acute and chronic kidney pathogenesis. The kidney, with its high energy demands, contains the second-highest mitochondrial content and oxygen consumption after the heart (Che et al. 2014; Duann and Lin 2017; Zhang et al. 2006). Perturbation of mitochondrial function—including impaired biogenesis, ATP depletion, and defective mitophagy—has been linked to kidney diseases such as acute kidney injury and chronic kidney disease (Che et al. 2014; Duann and Lin 2017). Oxidative stress, a hallmark of renal injury, is characterized by increased generation of ROS observed in both experimental models and patients with renal failure (Zhang et al. 2006). Oxidative damage arises from ischemia/reperfusion injury, energy deficits, and impaired clearance of damaged mitochondria (Duann and Lin 2017). The kidney’s role as the primary site for drug elimination renders it highly vulnerable to toxic injury by xenobiotics and their metabolites (Al-Naimi et al. 2019; Sharma and Singh 2023). Indeed, drug-induced nephrotoxicity accounts for nearly 20% of reported nephrotoxic events (Kim and Moon 2012; Sharma and Singh 2023).

GEN, an aminoglycoside antibiotic introduced in 1971, remains widely used for life-threatening gram-negative infections due to its potent bactericidal activity, low resistance rates, and cost-effectiveness (Arjmand et al. 2023; Plotnikov et al. 2013). Despite the availability of newer broad-spectrum antibiotics, aminoglycosides are still recommended for severe infections, including multidrug-resistant tuberculosis and certain zoonoses such as tularemia and plague (Huth et al. 2011). However, nephrotoxicity remains a major limiting factor in their clinical use. Gentamicin-induced nephrotoxicity (GIN) occurs in approximately 30–40% of treated patients, manifesting as non-oliguric renal failure and decreased glomerular filtration rate, typically a few days after therapy initiation (Abuelezz et al. 2016; Block and Blanchard 2022; López-Novoa et al. 2011; Shin et al. 2014). GEN exhibits poor tissue penetration except in the renal cortex, where about 5% of the administered dose accumulates in proximal tubular epithelial cells via megalin-mediated endocytosis (Dagil et al. 2013; Huth et al. 2011; Mathews and Bailie 1987; Morales et al. 2010). Within these cells, GEN persists for prolonged periods (half-life > 100 h), causing cytotoxic tubular injury (Dagil et al. 2013; Mathews and Bailie 1987). Following cellular uptake, GEN initially localizes to lysosomes. Upon exceeding threshold concentrations, lysosomal membranes rupture, releasing GEN into the cytosol, where it targets mitochondria (Arjmand et al. 2023; Dagil et al. 2013; Huang et al. 2020; Quiros et al. 2011). Due to structural similarities between bacterial and mitochondrial ribosomes and DNA, GEN disrupts mitochondrial translation and respiratory function, resulting in decreased ATP production and increased ROS generation (Boguszewska et al. 2020; Foster and Tekin 2016; Hong et al. 2015; Shulman et al. 2014; Min-Xin 2006). This oxidative stress triggers the intrinsic apoptotic pathway via cytochrome c release and caspase activation (Abuelezz et al. 2016; Adil et al. 2016; Cui et al. 2015; Negrette-Guzmán et al. 2015).

Preclinical studies provide strong evidence that mitochondrial dysfunction mediates GIN. The following sections summarize in vitro and in vivo studies investigating mitochondrial biomarkers and functional changes.

### Studies in vitro

Only a limited number of in vitro studies have directly addressed the impact of GEN on renal mitochondria, yet these investigations consistently indicate a mitochondrial mechanism (Table 1). The major findings of these studies are summarized below.

**Table 1:** Mitochondrial function markers in GEN-treated cells

Cell types	Mechanisms	References
Rat renal tubular epithelial cells	↑ Mitochondrial ROS generation and NADPH oxidase activity ↑ Expression of pro-apoptotic proteins (Bax, cytosolic cytochrome c, and caspase-9 and -3) ↓ The expression of anti-apoptotic protein (Bcl-2)	Shin et al. (2014)
Mouse proximal tubular cells	↑ Mitochondrial ROS generation ↓ mtDNA copy number ↓ Mitochondrial membrane potential	Yanagida et al. (2004)
Mitochondria isolated from renal tissue of rats	↑ Mitochondrial lipid peroxidation ↑ Release of iron in mitochondria	Chen et al. (2017)
Rat renal proximal tubular cells	↑ Mitochondrial ROS generation ↓ Mitochondrial membrane potential ↓ GSH levels ↑ GSSG and MDA levels ↑ Expression of pro-apoptotic proteins (caspase-3)	Arjmand et al. (2023)

↓ decreased, ↑ increased, *Bax* Bcl-2 associated X, *Bcl-2* B-cell lymphoma 2, *GEN* gentamicin, *GSH* glutathione, *GSSG* glutathione disulfide, *MDA* malondialdehyde, *mtDNA* mitochondrial DNA, *NADPH* nicotinamide adenine dinucleotide phosphate, *ROS* reactive oxygen species

Limited in vitro investigations corroborate this mitochondrial mechanism (Table 1). GEN exposure in renal tubular cells consistently elevates mitochondrial ROS, reduces membrane potential and glutathione (GSH) levels, and enhances pro-apoptotic signaling (Bax, cytochrome c, caspase-3/9), while suppressing anti-apoptotic B-cell lymphoma 2 (Bcl-2) (Arjmand et al. 2023; Chen et al. 2017; Shin et al. 2014). Interventions such as red ginseng extract, polyomavirus enhancer activator 3 (PEA3) overexpression, and mitochondrial transplantation attenuate these effects, suggesting therapeutic strategies that target oxidative stress and mitochondrial repair.

### Studies in vivo

In vivo studies have examined the effects of GIN on kidney mitochondria, further confirming the mitochondrial basis and providing indications for the mechanism. (Table 2). The studies were summarized below.

**Table 2:** Mitochondrial biomarkers and functional changes in GEN-treated animal models

Animal model	Duration of treatment	Treatment doses	Mechanisms	References
Female Sprague–Dawley rats	6 days, *ip*	100 mg/kg/day	↓ Levels/activity of GSH and Mn-SOD in mitochondria isolated from the renal tissue ↓ Mitochondrial ATP production capacity Opening of the mPTP ↑ Release of mitochondrial cytochrome c	Chiu et al. (2008)
Male Sprague–Dawley rats	7 days, *ip*	100 mg/kg/day	↓ Levels/activities of GSH, SOD, GR, GPx, and GST in mitochondria isolated from the renal tissue	Poon et al. (2008)
Male Wistar albino rats	8 days, *ip*	80 mg/kg/day	↑ Levels of total nitrate/nitrite and TBARS in renal tissue ↓ GSH levels in renal tissue ↓ ATP content and ATP/ADP ratio in renal tissues ↓ CoA-SH/acetyl-CoA ratio in mitochondria isolated from the renal tissue	Al-Shabanah et al. (2010)
Male rats	3, 6 days, *ip*	150 mg/kg/day	Opening of the mPTP ↑ Release of mitochondrial cytochrome c ↑ ROS generation in renal tissue ↓ Mitochondrial ETC complex-I and mitochondrial redox activities ↓ State 3 (ADP-stimulated) respiration rate in mitochondria isolated from the renal tissue	Morales et al. (2010)
Male Sprague- Dawley rats	7 days, *ip*	120 mg/kg/day	↓ Mitochondrial ETC complex (I, II, and IV) and mitochondrial redox activities ↓ Levels/activities of GSH, SOD, CAT, GR, and GPx in renal tissue ↑ TBARS levels and protein carbonyl content in renal tissue ↑ Levels/activity of TNF‐α, IL-6, NF-κB, and myeloperoxidase in renal tissue ↑ Expression of Bax, p53, and cleaved caspase 3 and caspase 9 in renal tissue ↓ Expression of Bcl-2 in renal tissue	Sahu et al. (2014)
Female Sprague–Dawley rats	6 days, *ip*	150 mg/kg/day	↓ GSH/GSSG ratio in mitochondria isolated from the renal tissue ↓ GR and IDH activities in mitochondria isolated from the renal tissue ↓ Mitochondrial ATP production capacity in mitochondria isolated from the renal tissue	Wong et al. (2014)
Female Sprague–Dawley rats	5 days, *ip*	100 mg/kg/day	↓ GSH levels and the GSH/GSSG ratio in mitochondria isolated from the renal tissue ↓ Activities of GR and IDH in mitochondria isolated from the renal tissue ↓ ATP levels in renal tissue ↓ Mitochondrial ATP production capacity	Chen et al. (2014)
Male Wistar rats	7 days, *ip*	75 mg/kg, every 12 h	Extensive damage in the mitochondrial morphology ↓ State 3 (ADP-stimulated) and state 4 (ADP-depleted) respiration rate in mitochondria isolated from the renal tissue ↓ mitochondrial ETC complex (I, II, and IV) activities Opening of the mPTP	Negrette-Guzmán et al. (2015)
Male Chinese Experimental Mini-pigs	10 days, i*m*	80 mg/kg/day	↑ Expression levels of some genes related to autophagy in renal tissue ↑ Expression levels of γ-H2AX and 8-OHdG in renal tissue ↑ Expression levels of carbonylated proteins and HNE in in renal tissue ↑ Expression of caspase-3 in renal tissue ↑ Expression levels of NF-κB in renal tissue Mitochondrial swelling and disintegration of cristae	Cui et al. (2015)
Male Sprague- Dawley rats	7 days, *ip*	120 mg/kg/day	↓ Mitochondrial ETC complex (I and IV) activities ↓ Levels/activity of GSH and SOD in renal tissue ↑ Expression of kidney injury molecule-1 and neutrophil gelatinase-associated lipocalin in renal tissue ↑ Expression of NF-κB in renal tissue ↓ Expression of Bcl-2 in renal tissue	Adil et al. (2016)
Male Wistar rats	14 days, *ip*	100 mg/kg/day	↓ Mitochondrial ETC complex (I, II, and IV) and mitochondrial redox activities ↓ ATP content in renal tissue ↑ MDA and NO levels in renal tissue ↓ Levels/activities of GSH, SOD, CAT, and GPx in renal tissue ↑ Expression of caspase-3 and Bax in renal tissue	Abuelezz et al. (2016)
Male Sprague- Dawley rats	10 days,* ip*	100 mg/kg/day	↓ Levels/activities of GSH, SOD, and CAT in renal tissue ↑ MDA levels in renal tissue ↑ ROS generation in mitochondria isolated from the renal tissue ↑ Mitochondrial membrane potential ↑ Release of mitochondrial cytochrome c mitochondrial swelling	Sepand et al. (2016)
Male Wistar rats	11 days, *ip*	60 mg/kg/day	↓ State 3 (ADP-stimulated) and state 4 (ADP-depleted) respiration rate in mitochondria isolated from the renal tissue ↓ Mitochondrial membrane potential ↓ Mitochondrial ETC complex-V activity Detected tetra-linoleoyl cardiolipin in mitochondria isolated from the renal tissue Mitochondrial swelling	Félix et al. (2017)
Wistar rats	14 days, *ip*	100 mg/kg/day	↓ Mitochondrial ETC complex (I, II, and V) activities ↑ MDA levels in renal tissue ↓ Levels/activities of GSH, SOD, and CAT in renal tissue ↑ TNF-α and IL-1β levels in renal tissue	Gumbar et al. (2023)
Male Sprague- Dawley rats	10 days, *ip*	100 mg/kg/day	↓ Levels/activities of GSH, SOD, CAT, and GPx in mitochondria isolated from the renal tissue ↑ TBARS and ROS levels in mitochondria isolated from the renal tissue ↓ IDH, α-KGDH, MDH, and SDH activities in mitochondria isolated from the renal tissue ↓ Mitochondrial ETC complex (I-IV) activities ↓ Mitochondrial membrane potential	Shahzadi et al. (2023)
Wistar rats	10 days, *ip*	100 mg/kg/day	↓ Mitochondrial ETC complex (I and V) activities ↑ TBARS and ROS levels in renal tissue ↓ GSH levels in renal tissue ↑ TNF‐α in renal tissue	Sangwan et al. (2024)

↓ decreased, ↑ increased, *8-OHdG* 8-hydroxy-2′-deoxyguanosine, *Acetyl-CoA* acetyl coenzyme A, *ATP* adenosine triphosphate, *ADP* adenosine diphosphate*, Bax* Bcl-2 associated X*, Bcl-2* B-cell lymphoma 2, *CAT* catalase, *CoA-SH* coenzyme A synthetase, ETC electron transport chain, *GEN* gentamicin, *GPx* glutathione peroxidase, *GR* glutathione reductase, *GSH* glutathione, *GSSG* glutathione disulfide, *GST* glutathione S-transferase, *H2AX* H2A histone family member X, *HNE* 4-hydroxy-2-nonenal, *IDH* isocitrate dehydrogenase, *IL-1β* Interleukin-1-beta, *IL-6* interleukin-6, *im* intramuscular, *ip* intraperitoneal, *MDA* malondialdehyde, *MDH* malate dehydrogenase, *Mn-SOD* manganese superoxide dismutase, *mPTP* mitochondrial permeability transition pore, *NF-κB* nuclear factor kappa-light-chain-enhancer of activated B cells, *NO* nitric oxide, *p53* tumor protein p53, *ROS* reactive oxygen species, *SDH* succinate dehydrogenase, *SOD* superoxide dismutase, *TBARS* thiobarbituric acid reactive substances, *TNF‐α* tumor necrosis factor-alpha, *α-KGDH* alpha-ketoglutarate dehydrogenase

Animal models confirm mitochondrial dysfunction as a key driver of GIN (Table 2). Repeated intraperitoneal or intramuscular administration in vivo results in:

- *Bioenergetic failure*: decreased ATP content, impaired ETC complex activities (I–IV), and reduced redox enzyme function (superoxide dismutase (SOD), GR, glutathione peroxidase (GPx)) (Abuelezz et al. 2016; Adil et al. 2016; Sahu et al. 2014).
- *Oxidative imbalance*: increased lipid peroxidation (malondialdehyde (MDA), TBARS), nitric oxide (NO) accumulation, and diminished GSH/Glutathione disulfide (GSSG) ratios (Al-Shabanah et al. 2010; Chen et al. 2014; Chiu et al. 2008; Poon et al. 2008; Sahu et al. 2014; Wong et al. 2014).
- *Structural injury*: mitochondrial swelling, cristae disintegration, and mPTP opening (Cui et al. 2015; Félix et al. 2017; Morales et al. 2010; Negrette-Guzmán et al. 2015).

A range of natural and synthetic compounds (e.g., Schisandrin B, naringin, metformin, curcumin, carvedilol) demonstrate protective effects by enhancing mitochondrial antioxidant capacity, stabilizing ETC function, and inhibiting apoptosis or promoting autophagy (Chiu et al. 2008; Félix et al. 2017; Morales et al. 2010; Negrette-Guzmán et al. 2015; Sahu et al. 2014).

Taken together, these studies delineate a unified pathogenic sequence: GEN accumulates in proximal tubular cells, localizes to mitochondria, disrupts electron transport and redox balance, induces ROS generation and ATP depletion, and culminates in intrinsic apoptosis and tubular injury. This mechanism is consistently observed across cell and animal models, highlighting mitochondrial biomarkers such as GSH/GSSG ratio, MDA, ETC complex activities, and cytochrome c release as hallmarks of toxicity. Importantly, interventions targeting oxidative stress, mitochondrial bioenergetics, or autophagy confer protection, suggesting mitochondria-focused strategies as promising avenues for mitigating aminoglycoside nephrotoxicity. Despite this convergence, significant gaps remain: clinical validation of mitochondrial biomarkers in patients is scarce, and translation of protective agents into human therapy has not yet been realized. Addressing these gaps could enable biomarker-guided monitoring and development of mitochondria-targeted therapeutics in nephrotoxic drug management.

## Therapeutic strategies targeting mitochondrial dysfunction in gentamicin-induced nephrotoxicity

Mounting evidence indicates that mitochondria serve not only as critical mediators of GIN but also as viable therapeutic targets. Numerous natural and synthetic compounds have demonstrated nephroprotective potential by modulating mitochondrial redox status, bioenergetics, or quality control mechanisms.

### Antioxidant and redox modulators

Accumulating experimental evidence highlights mitochondrial oxidative stress as a central mechanism underlying GIN. Accordingly, numerous antioxidant and redox-active compounds—such as Schisandrin B, naringin, ellagic acid, curcumin, metformin, the Yang-stimulating herbal formula VI-28, gymnemic acid, casticin, and β-sitosterol—have been investigated for their capacity to preserve mitochondrial homeostasis. Collectively, these agents counteracted GEN induced mitochondrial injury by suppressing ROS generation, lipid peroxidation, and protein oxidation, while restoring the activities of key antioxidant systems including GSH, SOD, catalase (CAT), and GPx (Chiu et al. 2008; Morales et al. 2010; Sahu et al. 2014; Sepand et al. 2016; Negrette-Guzmán et al. 2015; Poon et al. 2008; Gumbar et al. 2023; Shahzadi et al. 2023; Wong et al. 2014). By reinforcing the mitochondrial redox buffering network, these compounds stabilize the inner membrane, inhibit cytochrome c release, and ultimately suppress apoptotic signaling cascades in renal tubular cells.

Among the natural antioxidants, Schisandrin B has shown particularly robust nephroprotective efficacy, enhancing mitochondrial antioxidant capacity and maintaining bioenergetic competence, as evidenced by increased ATP production and retention of cytochrome c within mitochondria (Chiu et al. 2008). Similarly, naringin strengthened endogenous antioxidant defenses and reduced oxidative insult to lipids and proteins. Notably, it also mitigated mitochondrial-driven apoptosis by preserving organellar integrity and modulating the expression of pro- and anti-apoptotic proteins (Sahu et al. 2014). Ellagic acid exerted comparable protective effects, reducing mitochondrial ROS formation and swelling while preventing cytochrome c translocation into the cytosol, thereby maintaining mitochondrial membrane potential and structural fidelity (Sepand et al. 2016). In a parallel manner, curcumin preserved mitochondrial morphology and function through the stabilization of ETC complex activities, inhibition of mPTP opening, and maintenance of oxidative phosphorylation efficiency (Negrette-Guzmán et al. 2015).

Beyond phytochemicals, metformin has demonstrated a similar capacity to mitigate mitochondrial impairment. Specifically, it curtailed ROS overproduction, prevented mPTP opening and cytochrome c efflux, and sustained respiratory chain integrity and energy transduction (Morales et al. 2010). Likewise, VI-28 improved mitochondrial antioxidant status, thereby attenuating oxidative stress-driven mitochondrial injury and contributing to the preservation of renal function (Poon et al. 2008).

Gymnemic acid further protected renal mitochondria by reactivating ETC complexes, enhancing enzymatic antioxidant defense, and suppressing oxidative stress-induced cellular damage (Gumbar et al. 2023). In the same vein, casticin ameliorated bioenergetic failure by strengthening antioxidant capacity, stimulating the TCA cycle and ETC enzyme activities, and preserving mitochondrial membrane potential, which collectively mitigated oxidative and metabolic dysfunction (Shahzadi et al. 2023). Conversely, β-sitosterol exhibited negligible renoprotective efficacy despite its inherent antioxidant potential. GEN exposure markedly decreased the mitochondrial GSH/GSSG ratio, glutathione reductase (GR) and isocitrate dehydrogenase (IDH) activities, and ATP generation, indicating profound disturbances in mitochondrial redox homeostasis and oxidative phosphorylation. Consequently, β-sitosterol failed to prevent mitochondrial damage or promote compensatory autophagic responses (Wong et al. 2014).

Taken together, these findings reinforce the concept that antioxidant and redox modulators targeting mitochondrial dysfunction represent a promising therapeutic avenue for GIN. By maintaining redox equilibrium, safeguarding mitochondrial ultrastructure, and preserving energy metabolism, these compounds collectively attenuate the cascade of oxidative and apoptotic events driving gentamicin-induced renal injury.

### Bioenergetics enhancers

Agents like *L-carnitine, solanesol* and *ursolic acid* improve mitochondrial energy metabolism by restoring ATP production, correcting coenzyme A synthetase /acetyl-CoA ratios, and preserving ETC complex activities (I–V) (Al-Shabanah et al. 2010; Sangwan et al. 2024). By maintaining mitochondrial membrane potential and oxidative phosphorylation, these compounds limit bioenergetic failure, a key driver of tubular injury.

In a rat model of GIN, L-carnitine deficiency was associated with increased oxidative stress, impaired mitochondrial energy balance, and reduced ATP availability. Urinary carnitine loss and decreased renal tissue levels further indicated that carnitine depletion contributes to mitochondrial dysfunction (Al-Shabanah et al. 2010). Similarly, solanesol improved mitochondrial function and energy status in gentamicin-treated rats. Treatment reduced oxidative stress markers and restored parameters associated with normal energy metabolism, suggesting a protective effect on renal mitochondria (Sangwan et al. 2024). Moreover, ursolic acid isolated from *Herba Cynomorii* improved mitochondrial performance and energy production in the kidney. Treatment helped maintain cellular energy levels and overall mitochondrial function, indicating a protective role against gentamicin-induced damage (Chen et al. 2014).

Together, these findings suggest that agents targeting mitochondrial energy metabolism can alleviate GIN by sustaining mitochondrial function and preventing energy depletion in renal tissue.

### Autophagy and mitochondrial quality control modulators

Therapeutic interventions that enhance mitochondrial quality control, particularly mitophagy, have shown promise in mitigating GIN. Agents such as rapamycin and red ginseng promote the clearance of damaged mitochondria and thereby prevent excessive apoptotic and inflammatory signaling (Cui et al. 2015). Likewise, mitochondrial transplantation strategies have emerged as a potential approach to restore mitochondrial integrity and cellular homeostasis (Arjmand et al. 2023).

In a pig model of GIN, rapamycin was investigated for its role in regulating autophagic pathways, particularly mitochondrial autophagy. GEN administration markedly increased the expression of renal genes associated with autophagy, oxidative damage, and inflammation. Structural alterations such as mitochondrial swelling and cristae disintegration were also observed, indicating severe mitochondrial injury. The findings suggested that impairment of autophagic processes leads to accumulation of damaged mitochondria and exacerbation of renal injury (Cui et al. 2015). Similarly, mitochondrial transplantation in renal proximal tubular cells exposed to GEN demonstrated protective effects. Transplanted mitochondria restored mitochondrial function and reduced apoptotic activity, indicating that replenishing healthy organelles can effectively counteract mitochondrial dysfunction and cellular injury (Arjmand et al. 2023).

The protective potential of red ginseng extract was also demonstrated in GEN treated renal cells. Treatment reduced mitochondrial damage and attenuated both early and late apoptotic responses, suggesting its efficacy in preserving mitochondrial integrity under toxic stress (Shin et al. 2014). In addition, studies on PEA3 revealed that its overexpression conferred resistance to GEN induced mitochondrial dysfunction by preserving mitochondrial function and reducing oxidative damage, thereby highlighting a potential regulatory role in nephroprotection (Chen et al. 2017).

Collectively, these findings indicate that strategies aimed at enhancing mitochondrial autophagy, supporting mitochondrial renewal, or modulating mitochondrial regulatory pathways may represent promising therapeutic avenues for preventing or ameliorating GIN.

### Anti-apoptotic interventions

Several protective agents, including *carvedilol, berberine*, and *sitagliptin*, exert anti-apoptotic effects by downregulating pro-apoptotic markers (Bax, cleaved caspase-3/9) and upregulating Bcl-2, thereby interrupting the intrinsic apoptosis pathway triggered by mitochondrial injury (Abuelezz et al. 2016; Adil et al. 2016; Félix et al. 2017).

In an experimental model, carvedilol preserved mitochondrial function by improving respiratory parameters and preserving inner membrane integrity. It also reduced mitochondrial swelling and apoptotic activation by reducing cardiolipin peroxidation and subsequent cytochrome c release (Félix et al. 2017). Similarly, berberine ameliorated GEN induced mitochondrial dysfunction by stabilizing oxidative balance and downregulating pro-apoptotic mediators, including nuclear factor kappa-light-chain-enhancer of activated B cells (NF-κB) and Bax, while enhancing Bcl-2 expression. These effects collectively limited tubular injury and apoptotic progression (Adil et al. 2016). In line with these findings, sitagliptin demonstrated comparable mitochondrial protection by preserving ATP production, improving antioxidant defenses, and suppressing apoptosis-related proteins. Its action appears to prevent mitochondrial impairment and subsequent cell death signaling triggered by GEN exposure (Abuelezz et al. 2016).

Overall, these studies underscore the therapeutic potential of anti-apoptotic agents in maintaining mitochondrial stability and interrupting the intrinsic apoptosis pathway in GEN induced renal injury.

### Integrative perspective

Across preclinical studies, these strategies converge on three primary mitochondrial targets:

1. *Reduction of oxidative stress* (ROS scavenging, GSH replenishment)
2. *Restoration of bioenergetic function* (ETC complex stabilization, ATP synthesis)
3. .*Preservation of mitochondrial integrity and quality control* (mitophagy promotion, anti-apoptotic signaling)

Despite promising results, translation into clinical practice is limited by the lack of human studies and standardized biomarkers to monitor mitochondrial recovery. Future investigations should focus on validating these therapeutic targets in patient populations and exploring combinational approaches for enhanced efficacy.

## Conclusion

GEN, despite its potent antibacterial efficacy, is significantly limited by its nephrotoxic effects, which are primarily mediated by mitochondrial dysfunction. As mitochondria play a fundamental role in cellular energy metabolism, redox homeostasis, and apoptotic regulation, their impairment leads to severe cellular damage and renal injury (Arjmand et al. 2023; Yanagida et al. 2004). The structural and functional similarities between mitochondria and bacterial cells make mitochondria an unintended target of aminoglycoside antibiotics, leading to oxidative stress, mitochondrial dysfunction, and subsequent renal damage (Plotnikov et al. 2013). In line with these mechanisms, recent evidence demonstrates that gentamicin also perturbs mitochondrial dynamics—particularly by promoting excessive fission—and alters renal transporter expression, thereby intensifying apoptotic signaling and further aggravating renal injury (Zhang et al. 2025).

GEN accumulation in renal proximal tubular cells triggers a cascade of mitochondrial alterations, including excessive ROS generation, mtDNA damage, oxidative phosphorylation impairment, and the opening of the mPTP (Arjmand et al. 2023; Shin et al. 2014). These disruptions collectively result in ATP depletion, cytochrome c release, and activation of intrinsic apoptotic pathways, ultimately contributing to nephron loss and renal dysfunction (Cui et al. 2015; Morales et al. 2010). In addition to apoptotic pathways, high concentrations of GEN can also induce necrotic cell death via ATP depletion and lysosomal proteolysis, further aggravating kidney injury (Adil et al. 2016; Félix et al. 2017).

Importantly, cumulative evidence from experimental studies indicates that the extent of GIN and mitochondrial dysfunction is both dose- and duration-dependent, reflecting the antibiotic’s pharmacokinetic accumulation within renal proximal tubular cells. Repeated or prolonged exposure leads to progressive mitochondrial injury due to sustained intracellular retention of GEN, which disrupts oxidative phosphorylation, increases ROS production, and promotes apoptotic and necrotic pathways (Cui et al. 2015; Chiu et al. 2008). In chronic administration models, such as those described by Al-Shabanah et al. (2010) and Sangwan et al. (2024), extended GEN exposure resulted in marked reductions in ATP content, respiratory control ratio, and coenzyme A synthetase activity, indicating time-dependent mitochondrial bioenergetic collapse. Similarly, Chen et al. (2014) demonstrated that subchronic exposure produced sustained suppression of mitochondrial enzyme activities and antioxidant defenses, suggesting that the persistence of GEN within renal tissue amplifies oxidative and metabolic stress over time. Conversely, acute high-dose exposure models (Morales et al. 2010; Adil et al. 2016; Félix et al. 2017) revealed rapid onset of mitochondrial depolarization, ETC complex inhibition, and cytochrome c release, emphasizing a clear dose-dependent threshold beyond which abrupt bioenergetic failure occurs. The findings of Arjmand et al. (2023) further support that short-term but intense mitochondrial injury can develop even after single-dose exposure when cellular ROS and caspase activity exceed compensatory antioxidant capacity. Collectively, these studies delineate a biphasic toxicity pattern wherein high-dose acute exposure triggers immediate mitochondrial collapse and apoptosis, while lower but sustained exposure leads to cumulative mitochondrial damage, impaired mitophagy, and eventual necrosis. Thus, both the intensity and duration of GEN exposure critically shape the trajectory and severity of mitochondrial dysfunction, defining the temporal dynamics of renal injury progression.

Mitochondrial dysfunction is not the sole factor responsible for GIN. Recent studies indicate that endoplasmic reticulum stress, inflammation, autophagy impairment, and oxidative damage collectively exacerbate renal injury (Cui et al. 2015; Sahu et al. 2014; Sangwan et al. 2024). In particular, oxidative stress, characterized by an imbalance between ROS generation and antioxidant defense mechanisms, has been widely acknowledged as a key contributor to GEN induced renal damage (Chiu et al. 2008; Quiros et al. 2011) Excessive ROS generation results in lipid peroxidation, protein oxidation, and mitochondrial membrane potential disruption, thereby amplifying cellular damage (Chaves and Tadi 2023; Yanagida et al. 2004). Furthermore, GEN has been shown to directly interfere with mitochondrial ribosomes and mtDNA, leading to impaired mitochondrial gene expression and defective oxidative phosphorylation (Boguszewska et al. 2020; Hong et al. 2015). Given the pivotal role of mitochondria in this pathophysiology, targeted therapeutic strategies aimed at preserving mitochondrial integrity have garnered significant interest.

Several mitochondria-focused approaches have been explored to mitigate GIN. Antioxidant compounds such as naringin, ellagic acid, and ursolic acid-enriched extracts have demonstrated efficacy in reducing oxidative stress and preserving mitochondrial function (Chiu et al. 2008; Sepand et al. 2016; Wong et al. 2014). Additionally, pharmacological agents such as metformin, carvedilol, and sitagliptin have been found to inhibit the mPTP opening, restore mitochondrial respiratory chain activity, and prevent ATP depletion (Abuelezz et al. 2016; Gumbar et al. 2023; Morales et al. 2010). Another promising avenue is mitochondrial biogenesis stimulation via agents like rapamycin, which enhances autophagy-mediated clearance of damaged mitochondria, thereby improving renal outcomes (Cui et al. 2015). Moreover, modulation of mitochondrial dynamics has emerged as a therapeutic strategy; in particular, inhibition of excessive fission with the Drp1 inhibitor Mdivi-1 has been shown to attenuate acute kidney injury by regulating the NF-κB/JNK/SIRT3 signaling pathway (Gou et al.^2025^). Notably, mitochondrial transplantation has emerged as an innovative strategy, with evidence suggesting that the transfer of healthy mitochondria can restore cellular respiration and counteract GEN induced toxicity (Arjmand et al. 2023). However, these preclinical findings remain constrained by model dependency, limited pharmacokinetic characterization, and absence of long-term outcome evaluation. Translational advancement depends on clarifying how such interventions reach, integrate, and function within renal mitochondria in vivo.

Despite these advances, significant challenges remain in translating experimental findings into clinical practice. A key obstacle is the variability in mitochondrial function and susceptibility among individuals, influenced by genetic, metabolic, and environmental factors (Shin et al. 2014; Yanagida et al. 2004). Additionally, the complexity of mitochondrial interactions with other cellular organelles necessitates a comprehensive understanding of the crosstalk between mitochondria, the endoplasmic reticulum, and lysosomes in GIN (Chaves and Tadi 2023). Another critical aspect is the identification of early biomarkers for mitochondrial dysfunction, which would enable timely intervention and reduce the risk of irreversible renal damage (López-Novoa et al. 2011; Sharma and Singh 2023). To address these gaps and identify actionable mitochondrial signatures and therapeutic windows, future research needs to focus on developing mitochondria-targeted therapies, exploring combination treatment strategies, integrating mitochondrial protection into personalized medicine approaches, and integrative approaches combining systems biology, advanced imaging, and translational pharmacology.

In conclusion, mitochondria lie at the heart of GIN, serving as both primary targets of toxicity and key regulators of cell fate. While significant progress has been made in elucidating the molecular basis of injury, clinical translation remains limited. Future research should shift from descriptive strategies to mechanistic and biomarker-guided strategies. Understanding the complex mechanisms underlying mitochondrial dysfunction in kidney injury is crucial for developing effective nephroprotective interventions. Equally important is the rational design of aminoglycoside derivatives or formulations that maintain antimicrobial potential while minimizing mitochondrial interference. By bridging the gap between mechanistic research and therapeutic application, future studies may pave the way for novel strategies that mitigate renal complications while preserving the lifesaving efficacy of GEN. Combining mitochondrial-targeted therapies with sensitive diagnostic methods may enable early and personalized nephroprotection. While mitochondria-targeted therapies, including antioxidants, mPTP inhibitors, mitochondrial biogenesis stimulators, and mitochondrial transplantation, hold significant promise, their clinical application requires further validation through well-designed preclinical and clinical studies. Consequently, preserving mitochondrial integrity without diminishing the antibacterial efficacy of GEN is not only a pharmacological target but also a paradigm for safer antibiotic therapy.

## Data Availability

Not applicable for this article.

## Abbreviations

8-OHdG: 8-Hydroxy-2′-deoxyguanosine
Acetyl-CoA: Acetyl-coenzyme A
ADP: Adenosine diphosphate
ATP: Adenosine triphosphate
Bax: Bcl-2 associated X
Bcl-2: B-cell lymphoma 2
CAT: Catalase
CoA-SH: Coenzyme A synthetase
ETC: Electron transport chain
FADH_2_: Flavin adenine dinucleotide
GEN: Gentamicin
GIN: Gentamicin-induced nephrotoxicity
GPx: Glutathione peroxidase
GR: Glutathione reductase
GSH: Glutathione
GSSG: Glutathione disulfide
GST: Glutathione S-transferase
H2AX: H2A histone family member X
HNE: 4-Hydroxy-2-nonenal
IDH: Isocitrate dehydrogenase
IL-1β: Interleukin-1-beta
IL-6: Interleukin-6
im: Intramuscular
ip: Intraperitoneal
MDA: Malondialdehyde
MDH: Malate dehydrogenase
Mn-SOD: Manganese superoxide dismutase
mPTP: Mitochondrial permeability transition pore
mtDNA: Mitochondrial DNA
NADH: Nicotinamide adenine dinucleotide
NF-κB: Nuclear factor kappa-light-chain-enhancer of activated B cells
NO: Nitric oxide
PEA3: Polyomavirus enhancer activator 3
p53: Tumor protein p53
ROS: Reactive oxygen species
SDH: Succinate dehydrogenase
SOD: Superoxide dismutase
TBARS: Thiobarbituric acid reactive substances
TCA: Tricarboxylic acid
TNF-α: Tumor necrosis factor-alpha
α-KGDH: Alpha-ketoglutarate dehydrogenase
